# Supplementary material for: A large-scale retrospective study of opioid poisoning in New York State with implications for targeted interventions
Source: Sci Rep. 2021 Mar 4;11:5152. doi: 10.1038/s41598-021-84148-2 (PMC7933431; doi:10.1038/s41598-021-84148-2)
Supplement: Supplementary file 2 — Supplementary Information 1. [file 41598_2021_84148_MOESM2_ESM.docx]

**Title Page**

**Title**: A Large-Scale Retrospective Study of Opioid Poisoning in New York State with Implications for Targeted Interventions

**Abbreviated Title**: Trends and Patterns of Opioid Poisoning in New York State, 2010-2016

**Authors:**

Xin Chen Ph.D. ^1^, Wei Hou PhD^2^, Sina Rashidian MS^3^, Yu Wang BS^3^, Xia Zhao MS^4^, George Stuart Leibowitz PhD LICSW^7^, Richard N Rosenthal MD^5^, Mary Saltz MD^1,6^, Joel H Saltz MD PhD^1^, Elinor Randi Schoenfeld PhD^2^, Fusheng Wang PhD^1,3^

^1^ Department of Biomedical Informatics, Stony Brook University, Stony Brook, NY, USA

^2^ Department of Family, Population and Preventive Medicine, Renaissance School of Medicine, Stony Brook, NY, USA

^3^ Department of Computer Science, Stony Brook University, Stony Brook, NY, USA

^4^ School of Health Technology and Management, Stony Brook University, Stony Brook, NY, USA

^5^ Department of Psychiatry, Renaissance School of Medicine, Stony Brook, NY, USA

^6^ Department of Radiology, Renaissance School of Medicine, Stony Brook, NY, USA

^7^ School of Social Welfare, Stony Brook University, Stony Brook, NY, USA

**Corresponding Author:**

Fusheng Wang PhD

Department of Biomedical Informatics

Department of Computer Science

Stony Brook University

2313D Computer Science

Stony Brook, NY 11794-8330

Tel: (631)632-2594

Email: fusheng.wang@stonybrook.edu

**Conflict of Interest Disclosures:**

The authors disclose no conflicts of interest. This work was supported in part by grants ACI 1443054 and IIS 1350885 from the National Science Foundation, and an OVPR seed grant from Stony Brook University. The sponsors did not have any role in study design; collection, analysis, and interpretation of data; writing the report; or the decision to submit the report for publication.

**Financial disclosure:**

No financial disclosures were reported by the authors of this paper.

**Appendix**

| **Appendix Table 1. Standardized Regression Coefficients of Social Determinates for Opioid Poisoning Rate per 100,000 population at ZIP Code Level, NYS, 2010-2016** | | | | | | | |
| --- | --- | --- | --- | --- | --- | --- | --- |
| **Socio-economic Factors** | | **Overall OP Rate** | | **Heroin OP Rate** | | **Non-Heroin OP Rate** | |
|  |  | **Coefficient** | **P value** | **Coefficient** | **P value** | **Coefficient** | **P value** |
| Median Household Income, $US | | -0.235 | <.0001 | -0.096 | 0.001 | -0.252 | <.0001 |
| Race and Ethnicity | African American, % | -0.013 | 0.001 | -0.011 | 0.006 | -0.010 | 0.009 |
|  | Asian, % | 0.003 | 0.056 | 0.005 | 0.006 | 0.002 | 0.381 |
|  | Hispanic or Latino, % | 0.020 | 0.502 | 0.012 | 0.705 | 0.020 | 0.513 |
| Sex, male, % | | 0.010 | 0.687 | 0.028 | 0.270 | -0.003 | 0.903 |
| Education, High School graduate, % | | 0.053 | 0.074 | 0.043 | 0.151 | 0.044 | 0.136 |
| Housing units | | 0.009 | 0.804 | 0.072 | 0.043 | -0.030 | 0.388 |
| Population density (persons per square mile) | | -0.097 | 0.004 | -0.139 | <.0001 | -0.047 | 0.161 |

| Appendix Table 2. ICD-9 and ICD-10 Diagnosis Codes Related to Opioid Poisoning. Codes related to heroin are highlighted in bold. | | | |
| --- | --- | --- | --- |
| Years | **ICD Code** | **Version** | **Description** |
| 2010-2015 | 9650 | 9 | Poisoning; Opiates and Related Narcotics |
| 2010-2015 | 96500 | 9 | Poisoning; Opium (alkaloids), unspecified |
| 2010-2015 | **96501** | **9** | **Poisoning; Heroin** |
| 2010-2015 | 96502 | 9 | Poisoning; Methadone |
| 2010-2015 | 96509 | 9 | Poisoning; Other |
| 2010-2015 | **E8500** | **9** | **Accidental Poisoning; Heroin** |
| 2010-2015 | E8501 | 9 | Accidental Poisoning; Methadone |
| 2010-2015 | E8502 | 9 | Accidental Poisoning; Other Opiates and Related Narcotics |
| 2015 | T400X1A | 10 | Poisoning by opium, accidental (unintentional initial encounter) |
| 2015 | T400X1S | 10 | Poisoning by opium, accidental (unintentional), sequela |
| 2015 | T400X2A | 10 | Poisoning by opium, intentional self-harm, initial encounter |
| 2015 | T400X2S | 10 | Poisoning by opium, intentional self-harm, sequela |
| 2015 | T400X3A | 10 | Poisoning by opium, assault, initial encounter |
| 2015 | T400X3S | 10 | Poisoning by opium, assault, sequela |
| 2015 | T400X4A | 10 | Poisoning by opium, undetermined, initial encounter |
| 2015 | T400X4S | 10 | Poisoning by opium, undetermined, sequela |
| 2015 | T400X5A | 10 | Adverse effect of opium, initial encounter |
| 2015 | T400X5S | 10 | Adverse effect of opium, sequela |
| 2015 | **T401X1A** | **10** | **Poisoning by heroin, accidental (unintentional), initial encounter** |
| 2015 | **T401X1S** | **10** | **Poisoning by heroin, accidental (unintentional), sequela** |
| 2015 | **T401X2A** | **10** | **Poisoning by heroin, intentional self-harm, initial encounter** |
| 2015 | **T401X2S** | **10** | **Poisoning by heroin, intentional self-harm, sequela** |
| 2015 | **T401X3A** | **10** | **Poisoning by heroin, assault, initial encounter** |
| 2015 | **T401X3S** | **10** | **Poisoning by heroin, assault, sequela** |
| 2015 | **T401X4A** | **10** | **Poisoning by heroin, undetermined, initial encounter** |
| 2015 | **T401X4S** | **10** | **Poisoning by heroin, undetermined, sequela** |
| 2015 | T402X1A | 10 | Poisoning by other opioids, accidental (unintentional), initial encounter |
| 2015 | T402X1S | 10 | Poisoning by other opioids, accidental, sequela |
| 2015 | T402X2A | 10 | Poisoning by other opioids, intentional self-harm, initial encounter |
| 2015 | T402X2S | 10 | Poisoning by other opioids, intentional self-harm, sequela |
| 2015 | T402X3A | 10 | Poisoning by other opioids, assault, initial encounter |
| 2015 | T402X3S | 10 | Poisoning by other opioids, assault, sequela |
| 2015 | T402X4A | 10 | Poisoning by other opioids, undetermined, initial encounter |
| 2015 | T402X4S | 10 | Poisoning by other opioids, undetermined, sequela |
| 2015 | T402X5A | 10 | Adverse effect of other opioids, initial encounter |
| 2015 | T402X5S | 10 | Adverse effect of other opioids, sequela |
| 2015 | T403X1A | 10 | Poisoning by methadone, accidental (unintentional), initial encounter |
| 2015 | T403X1S | 10 | Poisoning by methadone, accidental (unintentional), sequela |
| 2015 | T403X2A | 10 | Poisoning by methadone, intentional self-harm, initial encounter |
| 2015 | T403X2S | 10 | Poisoning by methadone, intentional self-harm, sequela |
| 2015 | T403X3A | 10 | Poisoning by methadone, assault, initial encounter |
| 2015 | T403X3S | 10 | Poisoning by methadone, assault, sequela |
| 2015 | T403X4A | 10 | Poisoning by methadone, undetermined, initial encounter |
| 2015 | T403X4S | 10 | Poisoning by methadone, undetermined, sequela |
| 2015 | T403X5A | 10 | Adverse effect of methadone, initial encounter |
| 2015 | T403X5S | 10 | Adverse effect of methadone, sequela |
| 2015 | T404X1A | 10 | Poisoning by other synthetic narcotics, accidental, initial encounter |
| 2015 | T404X1S | 10 | Poisoning by other synthetic narcotics, accidental, sequela |
| 2015 | T404X2A | 10 | Poisoning by other synthetic narcotics, self-harm, initial encounter |
| 2015 | T404X2S | 10 | Poisoning by other synthetic narcotics, self-harm, sequela |
| 2015 | T404X3A | 10 | Poisoning by other synthetic narcotics, assault, initial encounter |
| 2015 | T404X3S | 10 | Poisoning by other synthetic narcotics, assault, sequela |
| 2015 | T404X4A | 10 | Poisoning by other synthetic narcotics, undetermined, initial encounter |
| 2015 | T404X4S | 10 | Poisoning by other synthetic narcotics, undetermined, sequela |
| 2015 | T404X5A | 10 | Adverse effect of other synthetic narcotics, initial encounter |
| 2015 | T404X5S | 10 | Adverse effect of other synthetic narcotics, sequela |

Appendix Video 1. Map animation of opioid poisoning (OP) patient rates per 100,000 population at county level, 2010-2016
